# Arterial Stiffness and Its Relationship to Cardiorespiratory Fitness in Children and Young Adults with a Fontan Circulation

**DOI:** 10.1007/s00246-019-02065-8

**Published:** 2019-02-15

**Authors:** Laurien C. M. Noortman, Eero A. Haapala, Tim Takken

**Affiliations:** 1 Faculty of Medicine, Utrecht University, Utrecht, The Netherlands; 2 Faculty of Sport and Health Sciences, University of Jyväskylä, Jyvaskyla, Finland; 3 Institute of Biomedicine, University of Eastern Finland, Kuopio, Finland; 4 Child Development and Exercise Center, Wilhelmina Children’s Hospital, P.O. Box 85090, 3508 AB Utrecht, The Netherlands; 5 Partner of Shared Utrecht Pediatric Exercise Research (SUPER) Lab, Utrecht, The Netherlands

**Keywords:** Arterial stiffness, Cardiorespiratory fitness, Fontan circulation, Univentricular heart, Youth, Pulse wave velocity, Augmentation Index

## Abstract

There are no previous studies on arterial stiffness and its associations with cardiorespiratory fitness in young Fontan patients. Therefore, we examined the arterial stiffness and its relationship to cardiorespiratory fitness in children and young adults with a Fontan circulation. Altogether, 17 Fontan patients and 26 healthy controls (16 females and 27 males aged 8–40 years) participated in this cross-sectional study. The cardiorespiratory fitness was assessed by cardiopulmonary exercise testing on a cycle ergometer and was defined as the standard deviation scores (SDS) of peak oxygen uptake per body mass (VO_2peak_/kg) based on the national reference values and assessed with cardiopulmonary exercise testing on a cycle ergometer. Aortic pulse wave velocity (PWV_ao_) as a measure of arterial stiffness and aortic Augmentation Index (AIX) as a measure of peripheral arterial tone, were assessed by non-invasive oscillometric device from upper arm. Body adiposity was determined by body mass index SDS and the sport participation by interview. Data were analyzed using linear regression analyses and Pearson’s correlations, adjusted for age and sex. Fontan patients had a lower VO_2peak_/kg-SDS (− 2.69 vs 0.078), higher PWV_ao_-SDS (1.13 vs − 0.24) and higher AIX (19.26% vs 8.49%) in comparison with healthy controls. PWV_ao_ and AIX were negatively associated with VO_2peak_/kg (standard regression coefficient (*β*) − 0.525, 95% confidence interval (CI) − 0.722 to − 0.227, *p* < 0.01 and *β* − 0.371, 95% CI − 0.672 to − 0.080, *p* = 0.014). Young Fontan patients have the arterial stiffness of healthy people who are twice as old. Thereby, children and young adults with a Fontan circulation have a lower cardiorespiratory fitness and less sport participation. Arterial stiffness is inversely associated with cardiorespiratory fitness and exercise training might be an intervention to improve vascular health in this population.

## Introduction

Approximately, 6–8 per 1000 children are born with a congenital heart disease (CHD) [1]. Fontan procedure is performed when there is congenitally one viable ventricle and a biventricular repair is not possible. Since the introduction of the Fontan procedure, most patients are able to survive into adulthood [2]. After this procedure, the blood from the superior and inferior vena cava is diverted to the pulmonary arteries, to partly repair the pulmonary blood flow [3]. Even when the Fontan procedure confers an improvement of ca. 20% in exercise tolerance, the patients still have a 30–40% lower exercise tolerance than healthy people [3]. In particular, peak oxygen uptake (VO_2peak_) and peak heart rate (HR_peak_) are impaired during physical activity [4]. Exercise is safe for stable Fontan patients and can be beneficial to increase exercise capacity [5]. Nevertheless, Fontan patients appear to be less engaged in physical exercise and this can have a negative impact on their vascular health [6].

The improvement in treatment of children with a CHD established that these patients can also reach the age at which the development of arteriosclerosis and its associated complications appear [2]. Arteriosclerosis is an important cause of the development of cardiovascular diseases and the process begins in early childhood [7]. Therefore, it is important to detect the development of arteriosclerosis at an early stage commence intervention and prevent cardiovascular morbidity and mortality.

The use of non-invasive measurements to determine the cardiovascular health of patients with a chronic disease has increased [8]. An impairment in the elasticity of the arterial walls is a sign of arteriosclerosis and a predictor of cardiovascular morbidity and mortality in the healthy population [9]. The aortic pulse wave velocity (PWV_ao_) can be used to measure the aortic stiffness and the PWV_ao_ tends to increase with age [10]. The aorta Augmentation Index is a surrogate marker of peripheral arterial tone and decreases with the development of arteriosclerosis in healthy people [11]. When the peripheral resistance is high, the AIX will increase, because a larger proportion of the pulse wave will reflect from the peripheral arteries [12]. A study in adult Fontan patients has noted an increase in arterial stiffness of these patients using non-invasive measurements [8].

A recent study from our group showed that chronically ill or physically disabled children and adolescents with decreased cardiorespiratory fitness have a stiffer aorta [13]. This has been partly explained by an increase in waist circumference. Another study concludes that cardiorespiratory fitness is related to PWV_ao,_ but not to AIX in healthy adolescents [14].

Little research has been conducted into the arterial stiffness, cardiorespiratory fitness and vascular problems in young Fontan patients. Therefore, the aim of this study is to analyze the arterial stiffness and the relationship with cardiorespiratory fitness in children and young adults with a Fontan circulation. We will also analyze if the arterial stiffness will increase by age in the Fontan patients.

## Methods

### Participants

In this study, the data were collected between October 2016 and August 2018. Subjects were recruited as a part of a larger study investigating the effects of altitude on exercise capacity (HYPOXIA study). Tests were performed at rest and during exercise. The children and adolescents of the study were recruited in the Netherlands by a shared Utrecht Pediatric Exercise Research lab (Super-Lab Utrecht) [15]. The exercise tests were performed in the Wilhelmina Children’s Hospital by an experienced exercise physiologist (Dr. T. Takken). 17 children and young adults with a Fontan circulation between the age of 8–40 were included. In addition, data from 26 healthy children and young adults between the age of 8–40 years were included. A subject was excluded from the study in case of an unstable cardiac function or fever. When the subject was not able to perform the exercise test on a cycle ergometer because of a mental or physical disability, he or she was also excluded from participation. Seven children or young adults were excluded, because the pulsations were not clear enough to measure the PWV_ao_ and AIX.

### Ethical Approval

The study was conducted in accordance with the Medical Research Involving Human Subjects Act (WMO) and Good Clinical Practice (GCP) and the principles of the Declaration of Helsinki. The participants of the study provided their informed consent before entering the study. When participants were under 18 years of age at the start of the study, their parents or caregivers had to sign the informed consent form. The medical ethics committee of the University Medical Centre Utrecht (Netherlands) approved the study.

### Assessment of Cardiorespiratory Fitness

The peak oxygen uptake (VO_2peak_) as measure of cardiorespiratory fitness was assessed by Cardio Pulmonary Exercise Testing (CPET) on an electronically braked upright cycle ergometer (Lode Corival, Groningen, The Netherlands). A RAMP incremental Godfrey protocol was used during the CPET [16]. The participant breathed through a facemask (Hans Rudolph, Inc. USA) connected to a calibrated metabolic cart (Geratherm Respiratory GmbH, Bad Kissingen, Germany). A gas analyzer and flow meter were used to measure oxygen (VO_2_) and carbon dioxide volume (VCO_2_) and breath-by-breath respiratory gas analyzes were performed during the test.

Children and young adults were verbally encouraged to perform the test until exhaustion. The exercise performance was at the maximum when there were subjective and objective signs of maximal effort (i.e. RER_peak_ (= VCO_2_/VO_2_) > 1.0, heart rate > 180/min, sweating, muscular fatigue, plateau of VO_2_).

The VO_2peak_/kg (ml/kg/min) was used to measure the cardiorespiratory fitness. The standard deviations scores (SDS) of the VO_2peak_ and the VO_2peak_/kg were calculated using the reference values of the healthy population in the Netherlands, adjusted for age and gender [17].

### Assessment of Body Size and Body Adiposity

Patient body height was measured by stadiometer (Prof Lange, Germany) and the body weight was measured by standard scale (Seca, Hamburg, Germany). The body mass (kg) was divided by body height squared (m^2^) to calculate the body mass index (BMI). The SDS’s of the weight, height and BMI were calculated using the reference values of The Netherlands [18].

### Assessment of Sports Participation

Amount of sport participation was accessed by interview and the children and adolescents were asked to determinate the amount of time a week they participate in sport. In this study, sport participation is defined as involvement in organized sports, but also daily exercise (i.e. biking to school, playing football with friends).

### Assessment of Arterial Stiffness

Systolic and diastolic blood pressures were measured using an automated cuff (SunTech Tango M2, SunTech Medical, USA). For the assessment of arterial stiffness, this study used the aortic pulse wave velocity (PWV_ao_). The peripheral arterial tone is measured by the Augmentation Index (AIX). These measurements were assessed by an arteriograph (TensioMed, Budapest, Hungary), a non-invasive oscillometric tonometry device. The method described by TensioMed was used correctly [19]. Research shows a strong association between the PWV_ao_ and AIX measured with the arteriograph and invasively obtained measurements (*R* > 0.9) [19]. A measurement of the PWV_ao_ with a SD greater than 1.0 m/s was considered as a disturbance in the metering and the PWV_ao_ was measured again. The SD to determinate the accuracy of the measuring was calculated by the algorithm of the software of TensioMed Arteriograph (Budapest, Hungary). The test was done at least twice a few minutes apart and the most accurate measurement was taken. When the participant could not be measured properly, the patient was excluded from the analysis. To define the SDS of the PWV_ao_ the reference values provided by the manufacturer were taken (Tensiomed, Budapest, Hungary). The aortic length was determined by the length between the sternal notch and the symphysis. The PWV_ao_ and AIX were calculated using the following equations [19]:

$${\text{PW}}{{\text{V}}_{{\text{ao}}}}={\text{aortic~length}}/({\text{reflection~time}}/2)\;({\text{m/s}})$$

$${\text{AIX}}=\left( {({\text{amplitude~of~the~reflection~wave}} - {\text{amplitude~of~the~direct~wave}})/{\text{pulse}}\;{\text{pressure}}} \right) \times 100~\;(\% )~.$$

### Statistical Methods

The independent-Samples *T* test was used to compare the baseline characteristics between boys and girls or controls and Fontan patients. No significant correlations were found between gender and the baseline characteristics, so the analysis of the data of the different genders were combined. Thereby, the baseline characteristics of the different groups (Fontan or control) were controlled to be corresponding. The baseline characteristics are presented as mean ± standard deviation or percentage.

The associations between the cardiorespiratory fitness, BMI, sport participation and PWV_ao_ or AIX were assessed using Pearson’s correlation. The correlation between PWV_ao_ or AIX and age were analyzed using linear regression analyzes. To correct for the age and gender, the SDS of the BMI, PWV_ao_, VO_2peak_ and VO_2peak_/kg were taken. The associations with the AIX or PWV_ao_ were controlled for systolic blood pressure.

Data were analyzed using the SPSS Statics, Version 25.0 (International Business Machines Corporation, Armonk, New York, United states of America). All tests were two-tailed, and values were considered statistically significant when the *p* value < 0.05.

## Results

### Baseline Characteristics

A total of 43 children and young adults comprised the study cohort; 17 Fontan patients and 26 healthy controls. There was no significant difference between the baseline data of the included and excluded group (*p* > 0.05). The gender distribution and average age between the Fontan patients and control were comparable, however, the BMI was lower for the Fontan patients (Table 1).

**Table 1:** Baseline characteristics

	All	Gender			Control or Fontan		
		Boys (*n* = 27)	Girls (*n* = 16)	*p* value	Control (*n* = 26)	Fontan (*n* = 17)	*p* value
Age (years)	21.0 ± 9.0	21.7 ± 9.7	19.8 ± 7.9	0.514	22.23 ± 9.0	19.12 ± 9.1	0.274
Fontan circulation (%)	39.5	40.7	37.5		–	–	
Boys (%)	62.8	–	–		61.5	64.7	
Sport participation (hours/week)	5.9 ± 3.2	6.1 ± 3.2	5.6 ± 3.1	0.660	6.9 ± 3.2	4.5 ± 2.6	0.016
Body height (cm)	168.5 ± 15.6	171.2 ± 15.8	163.9 ± 14.1	0.135	174.1 ± 13.3	159.9 ± 15.2	0.002
Body weight (kg)	59.2 ± 18.3	60.8 ± 19.1	56.5 ± 16.9	0.464	65.4 ± 17.2	49.7 ± 16	0.004
Body mass index (kg/m^2^)	20.3 ± 3.6	20.1 ± 3.5	20.7 ± 3.8	0.640	21.3 ± 3.4	18.96 ± 3.4	0.038
Body mass index SDS	0.11 ± 1.11	0.06 ± 1.13	0.19 ± 1.11	0.726	0.41 ± 0.93	− 0.33 ± 1.23	0.033
Systolic blood pressure in rest (mmHg)	132.5 ± 13.4	135.7 ± 13.0	127.1 ± 12.6	0.039	133.3 ± 10.9	131.2 ± 16.8	0.622
Diastolic blood pressure in rest (mmHg)	73.1 ± 10.2	74.5 ± 10.4	70.7 ± 9.7	0.242	72.6 ± 9.6	73.77 ± 11.4	0.728

The data are mean ± SD or percentages and the *p* values from the *t* test

### Fitness and arterial stiffness

In comparison to the control group, the Fontan patients have a significant lower VO_2peak_/kg (*p* = 0.0001), higher PWV_ao_ (*p* = 0.003) and higher AIX values (*p* = 0.0001) (Table 2).

**Table 2:** Fitness and arterial stiffness characteristics of the subjects

	All	Gender			Control or Fontan		
		Boys (*n* = 27)	Girls (*n* = 16)	*p* value	Control (*n* = 26)	Fontan (*n* = 17)	*p* value
VO_2peak_ (L/min)	2.3 ± 1.1	2.6 ± 1.3	2.0 ± 0.7	0.102	2.95 ± 1.03	1.40 ± 0.45	< 0.001
VO_2peak_ SDS	− 1.63 ± 2.49	− 1.72 ± 2.46	− 1.49 ± 2.60	0.767	− 0.39 ± 2.25	− 3.53 ± 1.40	< 0.001
VO_2peak_/kg (ml/kg/min)	38.6 ± 11.3	40.8 ± 11.6	34.9 ± 10.0	0.100	44.6 ± 8.8	29.4 ± 8.2	< 0.001
VO_2peak_/kg (ml/kg/min) SDS	− 1.02 ± 1.97	− 1.18 ± 2.05	− 0.73 ± 1.85	0.473	0.078 ± 1.46	− 2.69 ± 1.38	< 0.001
PWV_ao_ (m/s)	6.9 ± 1.36	7.0 ± 1.3	6.7 ± 1.4	0.623	6.4 ± 0.92	7.6 ± 1.61	0.003
PWV_ao_ SDS	0.30 ± 1.05	0.35 ± 1.06	0.22 ± 1.07	0.713	− 0.24 ± 0.68	1.13 ± 0.98	< 0.001
AIX (%)	12.8 ± 9.0	11.5 ± 10.2	14.9 ± 6.3	0.223	8.49 ± 6.10	19.26 ± 8.93	< 0.001

*SDS* standard deviation score, *PWV*_*ao*_ aortic pulse wave velocity, *AIX* aortic augmentation index (%), *VO*_*2peak*_ peak oxygen uptake

The data are mean ± SD or percentages and the *p* values from the *t* test

**Fig. 1:**
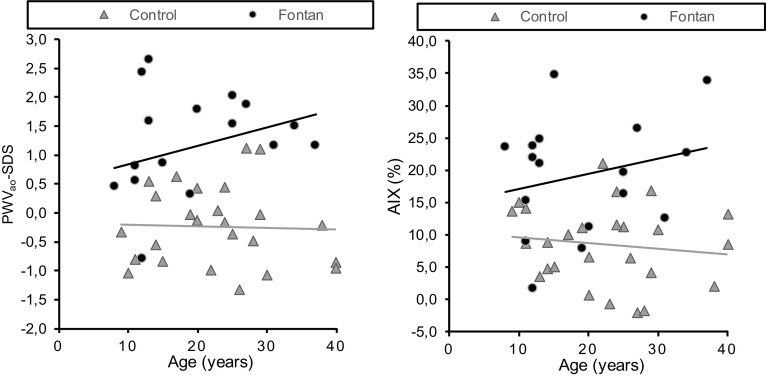
Relationship between arterial stiffness and age in Fontan patients. *PWV*_*ao*_ aortic pulse wave velocity, *AIX* Aortic Augmentation Index (%). PWV_ao_ is the standard deviation score compared to normal values for age and gender. The linear regression lines are showed for the subgroup of Fontan patients and the controls

In the Fontan patients, no significant correlation was found between age and AIX (*β* 0.235, 95% CI − 0.250 to 0.616 *p* = 0.364). However, the PWV_ao_ deviation of the Fontan patients tended to become larger with age (Fig. 1), but this correlation was not statistically significant (*β* 0.301, 95% CI − 0.088 to 0.644, *p* = 0.240). In the control group there was no correlation between AIX or PWV_ao_ with age.

### Associations of Cardiorespiratory Fitness with Arterial Stiffness

**Fig. 2:**
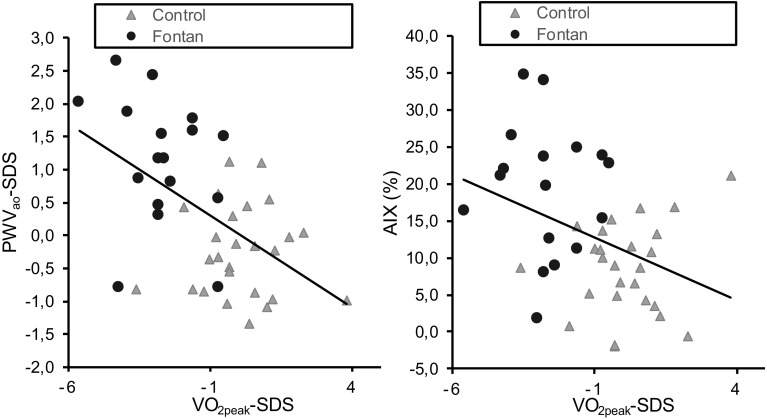
Correlation between arterial stiffness and cardiorespiratory fitness divided in subgroup of control and Fontan. *PWV*_*ao*_ aortic pulse wave velocity, *AIX* Aortic Augmentation Index (%), *VO*_*2peak*_*/kg* peak oxygen uptake per body mass. VO_2peak_/kg and PWV_ao_ are the standard deviations scores. The linear regression line is showed for the two groups (Fontan and controls) combined

A higher VO_2peak_/kg was significantly associated with a lower PWV_ao_ after normalizing for age and gender (Fig. 2). No significant relationship was found between the hours of sport participation or BMI and the PWV_ao_ (Table 3).

**Table 3:** Associations between aortic peak wave velocity and cardiorespiratory fitness, BMI and sport participation

	PWV_ao_					
	Combined (*n* = 43)			Fontan (*n* = 17)		
	*B*	95% CI	*p* value	*B*	95% CI	*p* value
VO_2peak_/kg	− 0.525	− 0.722 to − 0.227	< 0.001	− 0.279	− 0.698 to 0.362	0.278
BMI	− 0.150	− 0.491 to 0.232	0.343	0.110	− 0.398 to 0.490	0.673
Sport participation	− 0.211	− 0.466 to 0.090	0.186	− 0.084	− 0.586 to 0.396	0.748

The regression coefficients (*B*) and their 95% confidence intervals (CI) are showed for the standard deviations scores of VO_2peak_/kg, PWV_ao_ and BMI, adjusted for age and gender. The sport participation is in hours/week. The results are showed for all the participants (Fontan and control group combined) and for just the Fontan group

*VO*_*2peak*_*/kg* peak oxygen uptake per body mass, *PWV*_*ao*_ aortic pulse wave velocity, *BMI* body mass index

No significant association was found between age or gender and the AIX (respectively *p* = 0.678 and *p* = 0.223). After normalizing for age and gender, VO_2peak_/kg and BMI were negatively associated with AIX (Table 4). A higher amount of sport participation was associated with a lower AIX.

**Table 4:** Associations between aortic augmentation index (AIX), cardiorespiratory fitness, BMI and sport participation

	AIX					
	Combined (*n* = 43)			Fontan (*n* = 17)		
	*B*	95% CI	*p* value	*B*	95% CI	*p* value
VO_2peak_/kg	− 0.371	− 0.672 to − 0.080	0.014	− 0.065	− 0.411 to 0.230	0.804
BMI	− 0.438	− 0.668 to − 0.133	0.004	− 0.366	− 0.705 to 0.071	0.149
Sport participation	− 0.319	− 0.562 to − 0.029	0.042	− 0.184	− 0.607 to 0.222	0.479

The regression coefficients (*B*) and their 95% confidence intervals (CI) are showed for the standard deviations scores of VO_2peak_/kg and BMI, adjusted for age and gender. The sport participation is in hours/week. The results are showed for all the participants (Fontan and control group combined) and for just the Fontan group

*VO*_*2peak*_*/kg* peak oxygen uptake per body mass, *AIX* Aortic Augmentation Index (%), *BMI* body mass index

In the sub-analyzes of the Fontan group, no significant associations were found between VO_2peak_/kg per body mass, BMI or sport participation and the AIX nor with the PWV_ao_ (Tables 3, 4).

## Discussion

In our study, children and young adults with a Fontan circulation had a higher PWV_ao_ and AIX than the controls, indicating that the Fontan patients have stiffer arteries than healthy controls (Fig. 1). The mean PWV_ao_ of the patients with a Fontan circulation was 7.6 m/s and this correlates to a vascular age of 38–39 years, while the mean age of the patients was only 19 years. This finding indicates that Fontan patients must deal with the risk of cardiovascular diseases at a much younger age [7]. Other studies in patients children and adolescents with a Fontan circulation also conclude that Fontan patients have stiffer arteries [20–22].

Thereby, our results show that the deviation from the normal values becomes somewhat greater with age in the group of Fontan patients. This suggests not only that Fontan patients have the arterial stiffness of older arteries but also that the increase in stiffness during aging is more existent than in the control group. However, the correlation between age and a higher PWV_ao_ deviation and AIX as not significant, probably because of the low sample size. Nonetheless, other studies also found that young Fontan patients have a less deviant stiffness than older Fontan patients, which may suggest that the abnormalities arise with age [23, 24].

There might be a few reasons why Fontan patients have stiffer arteries. First of all, specific gene mutations are known that are involved in the heart malformation by CHD and some of these genes are important in the angiogenesis [20, 21]. Mutations in those genes might cause malformation of the arteries. Besides, patients with a Fontan circulation have a higher endothelin-1 level compared to healthy people [8]. Endothelin-1 has an important function in the pathophysiology of organ-injury after a cardiopulmonary bypass, by inducing proliferation, inflammation and vasoconstriction and might increase arterial stiffness [22, 24, 25]. Thereby, Fontan patients have a decreased concentration of nitric oxide, which causes reduction in flow-mediated dilatation of the arteries and the effect is greater after a longer cyanotic time before the procedure [26, 27]. Finally, the blood flow in the lungs and systemic circulation is impaired in Fontan patients [28], which changes shear stress and dyshomeostasis of the arteries [29]. The cardiac output is lower, and the arterial tonus increases by sympathetic activity to maintain a normal blood pressure, causing stiffer arteries [30].

The sample size of this study is too small to make a subgroup analyses for the Fontan patients to determine the relation between arterial stiffness and cardiorespiratory fitness. However, our results show a negative relationship between PWV_ao_ and cardiorespiratory fitness when the subgroups were combined. We observed no relationship with sport participation nor with BMI to PWV_ao_. The associations between a higher cardiorespiratory fitness or higher sport participation and a lower AIX were found. Few studies that used CPET to measure cardiorespiratory fitness and related VO_2peak_ to arterial stiffness. However, the inverse correlation between the arterial stiffness and the cardiorespiratory fitness are in line with studies in a population of healthy children and young adults [31, 32]. A study in young patients with a chronic disease or physical disabilities that used various exercise tests, suggests an association between the arterial stiffness and cardiorespiratory fitness, but they found waist circumference to be confounding [13]. In the present study, the waist circumference was not measured unfortunately. Other studies with a healthy population of children and young adults show a significant negative correlation between the cardiorespiratory fitness and the PWV_ao_, but this negative correlation was not found with AIX [14, 33, 34]. Meanwhile, another study in men without coronary heart diseases and a study in adults with a Fontan circulation show an association between AIX and cardiorespiratory fitness [8, 35]. A higher AIX in children is not always pathological, because growth and maturation might increase the AIX [36]. The AIX might be a good index of wave reflection caused by the peripheral arterial tone and the AIX tends to be more sensitive for natural fluctuation than the PWV_ao_ [37].

The impaired dilatation caused by arterial stiffening leads to exercise limitation, because the skeletal muscles receive less oxygen [38]. Thereby, studies show that a sedentary lifestyle is associated with endothelial dysfunction and arteriosclerosis [39]. Especially when the Fontan patients already have the arterial stiffness of someone who is twice their age, physical activity is important. Exercise and thereby an increase in shear stress, contribute to a better endothelial function and might help in reendothelialization [40]. Thus, it can be beneficial for the Fontan patients to be helped by creating a healthy and active lifestyle to improve endothelial function and the cardiorespiratory fitness [41]. The results can also be used for the development of therapeutic interventions to improve the arterial stiffness in Fontan patients.

The present study used a reliable and consistent method for assessing the PWV_ao_, AIX and cardiorespiratory fitness [19]. In addition, many other studies were compared with the present study to confirm the findings.

## Limitations

This cross-sectional study has some inherent limitations. The sample size of the study was too small to do reliable subgoup analyzes. Additional research with a bigger sample size is needed to draw robust conclusions for patients with a Fontan circulation. Furthermore, this study did not adjust for waist circumference, body fat percentage and fat-free mass, because some of the Fontan patients have a pacemaker and the body composition could not be measured by the bioelectrical impedance analysis. Other studies showed that those values might be associated with PWV_ao_ and AIX [13, 14]. The sport participation was assessed by asking how many hours a week the participant of the study used for sport, but no distinction was made between high and low intensity and daily physical activity (e.g. biking to school) was also included in this estimate. The AIX was not normalized for age and gender, however there were no significant correlations found in the data of our study between the AIX and age or gender.

We also recommend performing a follow-up study to monitor the longitudinal changes in vascular aging of patients with a Fontan circulation. To confirm our findings, the present study should be performed in a larger, multi-center population. In addition, longitudinal research could be done about the effect of a personalized sport program for children with a Fontan circulation on the arterial stiffness and cardiorespiratory fitness [41]. Finally, research must be conducted into whether vasodilators can be beneficial for the Fontan patients [42]. However, we consider the results of our study of clinical importance because of the large increase in vascular age.

## Conclusion

Children and young adults with a Fontan circulation have the arterial stiffness of healthy people who are twice as old. Thereby, young patients with Fontan circulation have a lower cardiorespiratory fitness and lower sport participation. A higher arterial stiffness is associated with a lower cardiorespiratory fitness. Exercise training to improve the cardiorespiratory fitness might be an intervention to improve vascular health in children and young adults with a Fontan circulation. Further research is needed to confirm these assumptions in Fontan patients.
